# Complementary Feeding Habits in Children Under the Age of 2 Years Living in the City of Adama in the Oromia Region in Central Ethiopia: Traditional Ethiopian Food Study

**DOI:** 10.3389/fnut.2021.672462

**Published:** 2021-10-28

**Authors:** Adugna Negussie Gudeta, Carin Andrén Aronsson, Taye Tolera Balcha, Daniel Agardh

**Affiliations:** ^1^Unit of Diabetes and Celiac Disease, Department of Clinical Sciences, Clinical Research Center, Lund University, Malmö, Sweden; ^2^Division of Clinical Infection Medicine, Department of Translational Medicine, Lund University, Malmö, Sweden

**Keywords:** children, complementary feeding, Ethiopia, gluten, malnutrition, teff

## Abstract

Updated information on child feeding practices, nutritional status, and trends related to parental sociodemographic variables is required in developing countries. The objective of this study was to describe infant feeding practices and associated sociodemographic factors among Ethiopian children with an emphasis on complementary feeding (CF). Information on infant feeding and anthropometric measures was obtained from 1,054 mother-child pairs participating in a birth cohort study of children born between 2017 and 2020 prospectively followed in the city of Adama located in the Oromia region of central Ethiopia. Logistic regression models were used to identify sociodemographic and food groups associated with the initiation of CF. The introduction of complementary foods at 6 months of age was 84.7% (95% CI, 82.5, 86.8). Vegetables, cereals (teff, wheat, barley), and fruits were most often the earliest types of foods introduced. Wasting, stunting, underweight, and low body mass index (BMI) by age were found in 6.0, 16.9, 2.5, and 6.3%, respectively. Maternal age and occupation were the factors associated with timely initiation of CF [*OR* = 2.25, (95% CI, 1.14, 4.41)] and [*OR* = 0.68, (95% CI, 0.48, 0.97)], respectively. This study demonstrates that the majority of Ethiopian children in the Oromia region follow the recommendations of WHO on CF.

## Introduction

The introduction of complementary foods during weaning at the age of 6 months is generally recommended to ensure the growth, health, and development of children to reach their optimal growth potential during the first 2 years of childhood (1). However, several studies have demonstrated that infant feeding practices are not followed worldwide (2, 3). Early or late introduction of adequate complementary foods of suboptimal quality and quantity, as well as poor hygienic conditions, is still common in developing countries.

Early and delayed introduction of complementary feeding (CF) may result in poor nutritional status and increased morbidity in growing children (4, 5). As such, the WHO Infant and Young Child Feeding (IYCF) practices strategy was developed, which includes infant feeding recommendations for breastfeeding children under the age of 2 years, the introduction of solid and semisolid foods at the age of 6 months, and a gradual increase in the amount and frequency of different foods as the child grows older (6). Inappropriate feeding practices attributed to poor nutritional knowledge can, therefore, be a significant contributor to poor nutritional status and growth development, as well as long-term risk of developing chronic diseases in developing countries (7–9).

Several studies from the sub-Saharan regions, which include Ethiopia, Malawi, Benin, and the Democratic Republic of Congo, have identified cereals as the most common source of food in early childhood, but access is highly dependent on sociodemographic factors with significant cultural differences (10–12). However, there is a lack of recent studies exploring the paradigm shift in dietary patterns of infants in African countries with increasing wealth since the millennium.

In Ethiopia, most of the solid foods first introduced to infants between 6 and 23 months of age are homemade, since commercially ready-made foods are often beyond the reach of the low-income population. The most common solid foods are based on three major staples that are locally available, including (1) corn*/*enset*/*teff, (2) wheat/barley, and (3) sorghum/maize, all of which meet the nutritional contents recommended by WHO for children between 6 and 23 months of age (Supplementary Table 1) (13). Homemade solid foods for infants in Ethiopia are mainly based on cereals or starchy tubers such as corn (*Zea mays*), sorghum (*Sorghum biocolor*), millet (*Panicum miliaceum*), oats (*Avena sativa*), teff (*Eragrostis tef* ), rice (*Oryza sativa*), yam (*Dioscorea*), potato (*Solanum tuberosum*), barley (*Hordeum vulgare*), and legumes (*Fabaceae*). These foods are generally served as gruel, porridge, fetfet, kitta, and bread. However, the consumption of meat (beef, lamb, goat, poultry), fruits, and vegetables is very low (13).

Teff (*Eragrostis tef* ) is the most common staple food in Ethiopia and is an important part of the cultural heritage and national identity (14). Although teff is considered to be highly nutritious (15, 16), the 2019 Ethiopia Demographic and Health Survey (EDHS) report found that about 37% of children younger than 5 years of age were stunted (of whom 12% were severely stunted), 7% were wasted (of whom 1% were severely wasted), and 21% of the children were underweight (of whom 6% were severely underweight). The EDHS nutritional survey also found that undernutrition differed between regional location, sex, and age groups of children (17).

The aim of this study was to describe recent infant feeding practices and associated sociodemographic factors in children under the age of 2 years who were born between 2017 and 2020 and lived in the city of Adama and the surrounding Oromia region in central Ethiopia.

## Materials and Methods

### Study Population

The main aim of the Traditional Ethiopian Food (TEF) study is to investigate the genetic propensity, feeding practices, nutritional status, and gut microbiome on the risk of celiac disease in children living in the Oromia region in central Ethiopia (18). Between 2017 and 2020, 1,389 newborn children were approached from the general population of the city of Adama and the surrounding Oromia region of central Ethiopia to be enrolled in a 4-year follow-up study. The cohort consisted of women and their offspring participating in parallel research studies at three public health facilities in the city of Adama. Mothers were participating in the pregnancy tuberculosis study investigating the role of tuberculosis infection on pregnancy outcomes, while infants were participating in the TEF birth cohort study examining the prevalence of celiac disease in children (18, 19). A physical examination was performed, and baseline data were collected when the child was 6 weeks of age and then subsequently at 9, 18, 24, 36, and 48 months of age.

### Food Questionnaire Data

At the scheduled study visits, a legal guardian filled in structured questionnaires regarding the information on the early infant feeding practices of the participating child. The form was developed in English and translated to the local language used for the data collection. The food questionnaire was developed for this study to gather information on breastfeeding, time for the introduction of complementary foods, and dietary infant feeding habits.

For the present study, information about breastfeeding duration and age at first introduction to complementary foods was examined for 1,054 children whose mothers had completed the questionnaire at the 9 and 18 months of age visits. Baseline maternal sociodemographic data were collected during the antenatal care (ANC) follow-up of the mother and at the 6-week post-delivery visit for the pregnancy tuberculosis cohort (19).

### Anthropometrics

Anthropometric measurements were done by trained nurses according to the WHO manual. Children were measured while lying down without wearing shoes using a calibrated length board for height. The weight of the children was measured with lightweight clothing and recorded to the nearest 0.1 kg using an infant weight scale. A Z-score <-2 SD in height-for-age was defined as stunted, <-2 SD in weight-for-height was defined as wasted, <-2SD in weight-for-age was defined as underweight, and <-2 SD in body mass index (BMI) for age was defined as low BMI (20).

### Statistical Methods

Frequency distribution and descriptive characteristics were investigated for maternal age, marital status, education, occupation, family size, as well as the birth order, sex, and age of the child at the introduction of selected solid foods. Logistic regression models were used to identify sociodemographic variables associated with the timing of complementary food initiation. After the necessary adjustments, adjusted odds ratios (AOR) with 95% confidence intervals (CI) were used to assess the strength of association. *P*-values <0.05 were considered statistically significant. Data were analyzed using the Statistical Package for the Social Sciences (SPSS) software for Windows (version 25; SPSS Inc. Chicago, IL). Anthropometric data were analyzed by using WHO Anthro (version 3.2.2) and supported by SPSS.

## Results

### Sociodemographic Characteristics of the Study Population

A total of 1,054 mother-child (53.5% boys) pairs from the 9- and 18-month visits were included in the study (Table 1). The median age of mothers in the study was 25 (range 15–40) years. The distribution of the marital status of mothers showed that the majority of women (96.5%) were married. More than half of the mothers (68.8%) had 6 years of primary school education or longer. Most of the women identified their religious affiliation as Orthodox Christianity (60%), were housewives by occupation (63.8%), and had an average of three family members living in the household (72.6%). Information about the mode of the delivery indicated that most mothers gave birth by vaginal delivery (84.4%). A third of the participating infants (33.6%) was the first-born child, and the median age of the children at the time of data collection was 9 months (range 9–18).

**Table 1:** Proportion of initiation of complementary feeding (CF) at 6 months of age by maternal sociodemographic characteristics.

**Variable**	**N (%)**	**Initiation of CF at 6 months of age**	
		**Yes (%)**	**No (%)**
**Maternal age:**			
<20	161 (15.3)	144 (89.4)	17 (10.6)
20–24	259 (24.6)	213 (82.2)	46 (17.8)
25–30	443 (42.0)	374 (84.4)	69 (15.6)
>30	189 (17.9)	152 (80.4)	37 (19.6)
Missing data	2 (0.2)		
**Marital status:**			
Married	1017 (96.5)	852 (83.8)	165 (16.2)
Single	35 (3.3)	31 (88.6)	4 (11.4)
Missing data	2 (0.2)		
**Family size:**			
≤ 3	762 (72.6)	641 (84.1)	121 (15.9)
≥ 4	288 (27.3)	241 (83.7)	47 (16.3)
Missing data	11(1.0)		
**Maternal education:**			
<6th grade	326 (30.9)	270 (82.8)	56 (17.2)
≥6th grade	726 (68.9)	613 (84.4)	113 (15.6)
Missing data	2 (0.2)		
**Maternal working condition:**			
Housewife	668 (63.4)	574 (85.9)	94 (14.1)
Employed	382 (36.2)	307 (80.4)	75 (19.6)
Missing data	4 (0.4)		
**Mother's religious affiliation:**			
Orthodox	631 (59.9)	522 (82.7)	109 (17.3)
Muslim	154 (14.6)	127 (82.5)	27 (17.5)
Protestant Christian	263 (25.0)	230 (87.5)	33 (12.5)
Missing data	6 (0.5)		
**Mother's first child:**			
Yes	354 (33.6)	300 (84.7)	54 (15.3)
No	700 (66.4)	585 (83.6)	115 (16.4)
**Mode of delivery:**			
Vaginal	890 (84.4)	747 (83.9)	143 (16.1)
Cesarean section or other	130 (12.3)	114 (87.7)	16 (12.3)
Missing data	34 (3.2)		
**Child's sex:**			
Male	564 (53.5)	466 (82.6)	98 (17.4)
Female (reference)	490 (46.5)	419 (85.5)	71 (14.5)
**Breastfeeding:**			
Yes	915 (88.2)	772 (84.4)	143 (15.6)
No	122 (11.8)	98 (80.3)	24 (19.7)

### Infant Feeding Practices and Factors Associated With Early CF

**Figure 1:**
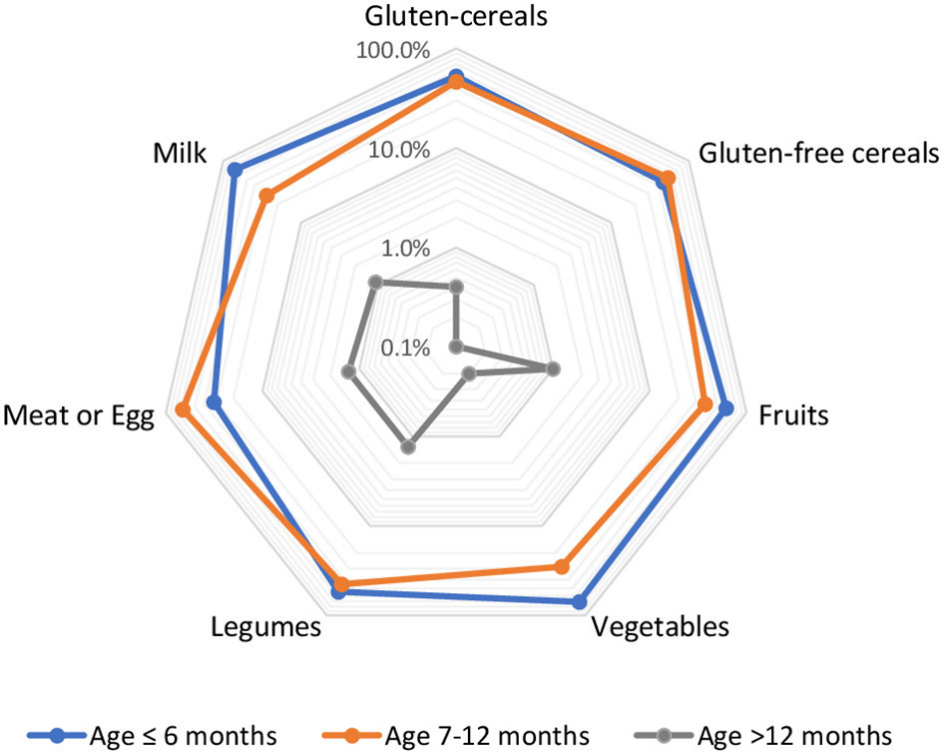
First introduction to complementary foods by age (%).

At the age of 6 months, 86.1% of the children were still being breastfed and had started CF. The majority (84.0%) of children were introduced to solid, semi-solid, or liquid forms of complementary food, on an average at 6 months of age. Few children (3.5%) were introduced to complementary food before 6 months of age. A cow milk-based infant formula was among the first foods to be introduced, often initiated earlier than 2 months by some families. The most common foods introduced before 6 months were milk, vegetables, fruits, gluten-containing cereals, legumes, gluten-free cereals, while meat or eggs were the most common foods introduced at 6 months of age (Figure 1).

Gluten-containing cereals (wheat, barley, and rye) were introduced at a median age of 6.8 months (range 4–18), while a gluten-free cereal such as teff was introduced at a median age of 7.0 (range 5–16) months. The majority of children were fed with both gluten-containing foods (53.0%) and gluten-free cereal-based foods (52.0%) more than two times a day. Vegetables, fruits, gluten-containing grains, legumes, and milk were often introduced simultaneously at 6 months, whereas teff, meat, and eggs were introduced, on average, after 6 months of age.

Young maternal age ( < 20 years) was associated with timely initiation of CF (i.e., by age of 6 months) as compared to older mothers (> 20 years) [*COR* = 2.06, (95% CI, 1.11, 3.83) and *AOR* = 2.66, (95% CI, 1.28, 5.53)]. Mothers who were employed outside of the home were also associated with the time of initiation of CF compared with mothers who reported their occupation as a housewife [*COR* = 1.49, (95% CI, 1.07, 2.08) and *AOR* = 0.68, (95% CI, 0.48, 0.97)] (Table 2). None of the other sociodemographic factors (maternal education, marital status, religion, the birth order of the child, or gender) were associated with the timing of CF in this study.

**Table 2:** Multivariable logistic regression analysis of factors associated with the initiation of CF at 6 months of participating children.

**Variable**	**COR (95%CI)**	**AOR (95%CI)**
**Maternal age:**			
<20	2.06 (1.11–3.82)^*^	2.66 (1.28–5.53)^*^
20–24	1.13 (0.70–1.18)	1.18 (0.68–2.03)
25–30	1.32 (0.85–2.05)	1.38 (0.86–2.23)
>30 (reference)	1	1
**Marital status:**			
Married	1	1
Single	1.50 (0.52–4.31)	1.58 (0.54–4.64)
**Family size:**			
≤ 3 (reference)	1	1
≥ 4	0.97 (0.67–1.40)	1.18 (0.76–1.82)
**Maternal education:**			
<6th grade	0.89 (0.63–1.26)	0.93 (0.64–1.37)
≥6th grade	1	1
**Maternal occupation:**			
Housewife (reference)	1	1
Employed	1.49 (1.07–2.08)^*^	0.68 (0.48–0.97)^*^
**Mother's religious affiliation:**			
Orthodox (reference)	1	
Muslim	0.08 (0.45–1.04)	-
Protestant Christian	0.68 (0.39–1.17)	-
**Mother's first child:**			
Yes	1.09 (0.77–1.55)	0.98 (0.65–1.50)
No (reference)	1	1
**Mode of delivery:**			
Cesarean section	0.73 (0.42–1.28)	0.98 (0.65–1.50)
Vaginal	1	1
**Child's sex:**			
Male	0.73 (0.42–1.28)	1.24 (0.87–1.76)
Female (reference)	1	1
**Breastfeeding:**			
Yes	0.76 (0.47–1.22)	0.71 (0.43–1.18)
No	1	1

* *P < 0.05; CF, complementary food; CI, confidence interval; AOR, adjusted odds ratio; COR, crude odds ratio*.

### Anthropometrics

**Figure 2:**
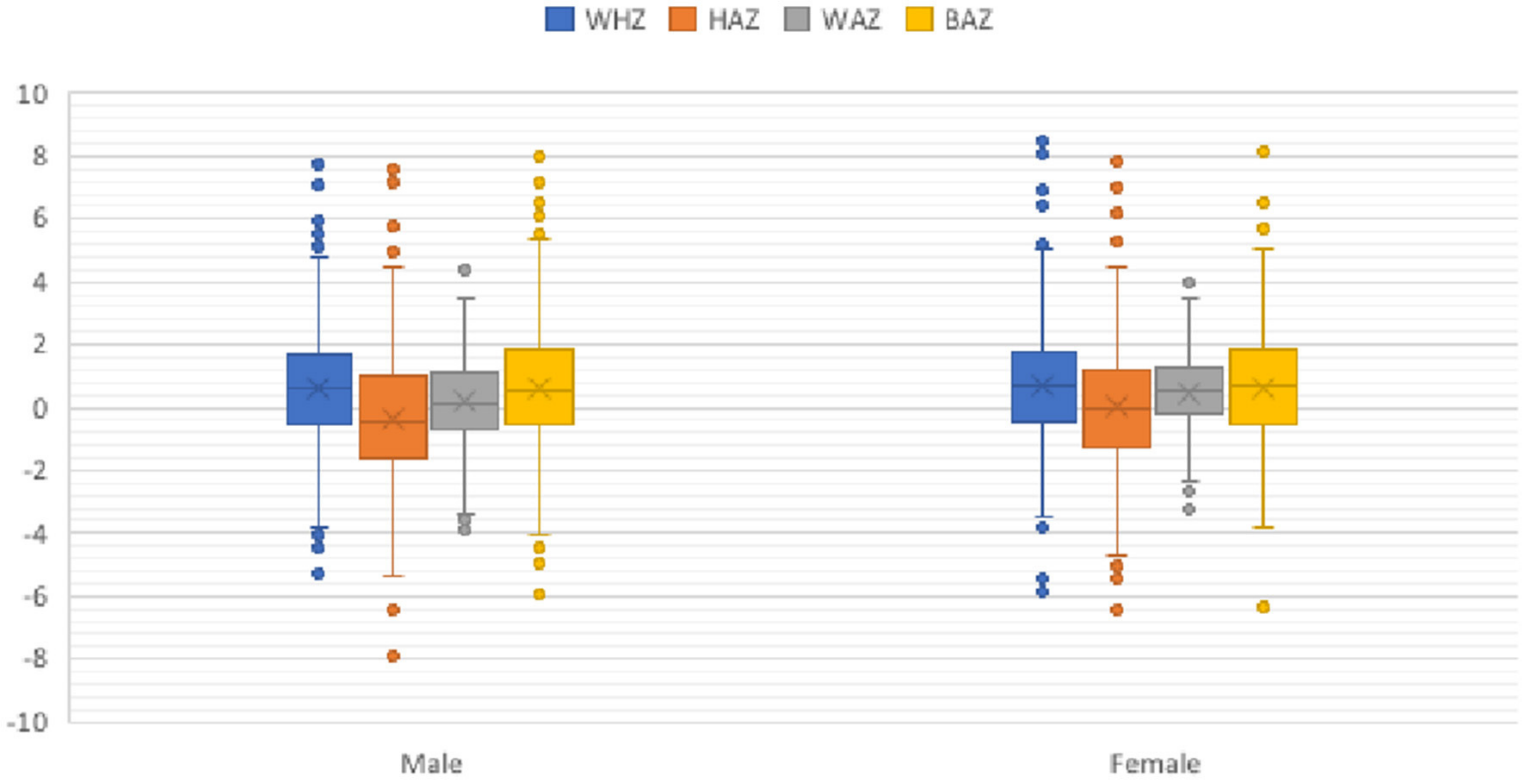
Child anthropometrics by sex. BAZ, body mass index for age z-score (<-2 SD, low BMI); HAZ, height-for-age z-score (<-2 SD, stunted); WAZ, weight-for-age *z*-score (<-2 SD, underweight); WHZ, weight-for-height z-score (<-2 SD, wasted).

The child anthropometry revealed that about 16.9% of the children were stunted (Figure 2). The corresponding numbers for wasting, underweight, and low BMI by age were 4.5, 2.5, and 6.3%, respectively (Figure 2). There was an association between a child's sex with stunting (95% CI, 0.12, 0.63) and underweight (95% CI, 0.08, 0.036), respectively (Figure 2).

## Discussion

The first 2 years of life are a critical time window for optimal growth, development, and health of a child (21). The introduction of an adequate, appropriate, and safe dietary intake at the age of 6 months following exclusive breastfeeding is recommended by WHO since inadequate nutrient intake during this period increases the risk for underweight or overweight with potentially lifelong health problems.

The present study included children under the age of 2 years living in the city of Adama and the surrounding area in the Oromia region in central Ethiopia. The median age of initiation of CF was 6 months of age and only 3.6% of the parents started CF earlier than the WHO recommendations (22). Results of this study are in line with a study conducted in Addis Ababa, in which 83% followed the WHO infant feeding recommendations (10). On the other hand, the proportion of children following the recommendations is higher than studies conducted in other parts of the country, including Mekele in northern Ethiopia (62%) (23), Arsi Negelle (72%) (24), and in Sodo town in southern Ethiopia (71%) (25). The proportion is also much higher than the finding from the national prevalence in Ethiopia (56%) (26), Tahatay Maichew (53%) (27), Gonder town in northern Ethiopia (47%) (28), Damot Weydie (50.6%) (29), and Kamba in southern Ethiopia (40.6%) (30).

Disparities in the timely initiation of CF between different parts of Ethiopia could be explained by the variation in maternal occupations, awareness of recommended WHO guidelines, urban vs. rural residency of the study participants, data collection approaches, the identified religious affiliation of a population, and/or socioeconomic status. Location can also contribute to varied access to health services and information. All mothers who were enrolled in the present study attended both ANC and postnatal care at health institutes where the study was conducted. The variables are both important indicators of successful infant and young children feeding practices.

The majority of children in the study received foods made from vegetables (96%) and gluten-free cereals (92%) by 18 months of age. In contrast, foods made from legumes and dairy products are received by relatively low numbers of children. These results are in accordance with previous studies in Ethiopia regarding foods made from cereals and dairy products but are higher than other studies, which usually found low consumption of vegetables (31). Discrepancies in types of foods consumed might be explained by a variation in the socioeconomic status of the study participants or differences among parents' awareness of the importance of the variety of food items recommended by WHO.

Being a mother younger than age 20 years was one of the factors associated with following the complementary infant feeding recommendations of WHO, which is in contrast with studies from Southwest of Ethiopia (30). The other associated factor with the time of infant feeding was maternal occupation (32). These findings are in contrast with those of previous studies that demonstrated an association between time of infant feeding and religious affiliation (33) or mode of delivery (34, 35). The disparities in CF habits in mothers by age compared with previous studies from other regions of Ethiopia may be severalfold; this study indicates the associations with the employment of the mother outside the home and having access to health services during ANC and postnatal care.

In the present study, a lower proportion of the children experienced wasting, stunting, underweight, and low BMI for their age compared with studies from other areas of Ethiopia (26, 36). This discrepancy might be related to the difference in the culture of feeding, parents' awareness of WHO feeding recommendations, or socioeconomic variation, access to information or methods of measures on IYCF practices. Another plausible explanation could be a selection bias in study participants, in which those attending health services are also more likely to participate in a research study. Although these may all confound the findings, stunting and underweight were more common in males compared with females, which confirms previous reports (37, 38).

A strength of the present study is the study design in which data were continuously and prospectively collected close to birth with information about the health status of both the mother and the child. A limitation is that data were collected in only one city with close surroundings, which may not be representative for all surrounding rural areas or other urban cities of Ethiopia and, therefore, may not fully represent the community in the Oromia region. Moreover, information on CF habits was only presented at the 9- and 18-month visits, which may not be represented as the children grow older. Other potential limitations include missing data on the socioeconomic status of the mother and only including one child per family to participate in the study. It cannot be ignored that CF habits differ in families with more than one child in the household and families with different socioeconomic backgrounds. Also, the study focuses on the age of initiation of CF practices and does not investigate CF indicators, such as minimum dietary diversity, meal frequency, and recommended diets. Finally, the quality and quantity of the food types were not investigated.

## Conclusion

In conclusion, the present study from the city of Adama in the Oromia region in central Ethiopia demonstrates that a majority of mothers follow the WHO recommendations in introducing CF to infants at the age of 6 months. Gluten-free cereals are among the most consumed food types by children in the study area. Mothers younger than 20 years old and the employment of mothers outside of the home were factors associated with the timely initiation of the CF of the infant. This finding is noteworthy since it gives updated information on infant feeding and stimulates more research into the frequency, quality, and quantity of food consumed by infants.

## Data Availability Statement

The original contributions presented in the study are included in the article/Supplementary Material, further inquiries can be directed to the corresponding author.

## Ethics Statement

The studies involving human participants were reviewed and approved by Armauer Hansen Research Institute, National Research Ethics Committee of Ethiopia, Lund University. Written informed consent to participate in this study was provided by the participants' legal guardian/next of kin.

## Author Contributions

AG conducted the research and statistical analysis and drafted the manuscript. DA and CA contributed to the result interpretation and writing of the manuscript. DA contributed to the study design, editing of the manuscript, and overall supervision of the study. CA and TT contributed to the critical reading and editing of the manuscript. All authors accepted the final version of the manuscript.

## Funding

This research was aided by the funds provided by the SUS Stiftelser och Fonder, Region Skåne FoU-medel, Swedish Celiac Disease Foundation, Håkanssons Stiftelse, and Pålssons Stiftelse.

## Conflict of Interest

The authors declare that the research was conducted in the absence of any commercial or financial relationships that could be construed as a potential conflict of interest.

## Publisher's Note

All claims expressed in this article are solely those of the authors and do not necessarily represent those of their affiliated organizations, or those of the publisher, the editors and the reviewers. Any product that may be evaluated in this article, or claim that may be made by its manufacturer, is not guaranteed or endorsed by the publisher.
