# Supplementary material for: Complementary Feeding Habits in Children Under the Age of 2 Years Living in the City of Adama in the Oromia Region in Central Ethiopia: Traditional Ethiopian Food Study
Source: Front Nutr. 2021 Oct 28;8:672462. doi: 10.3389/fnut.2021.672462 (PMC8581346; doi:10.3389/fnut.2021.672462)
Supplement: Supplementary file 1 [file Table_1.DOCX]

**Supplementary table**. Appropriate foods for complementary feeding according to WHO recommendations (WHO, 2009).


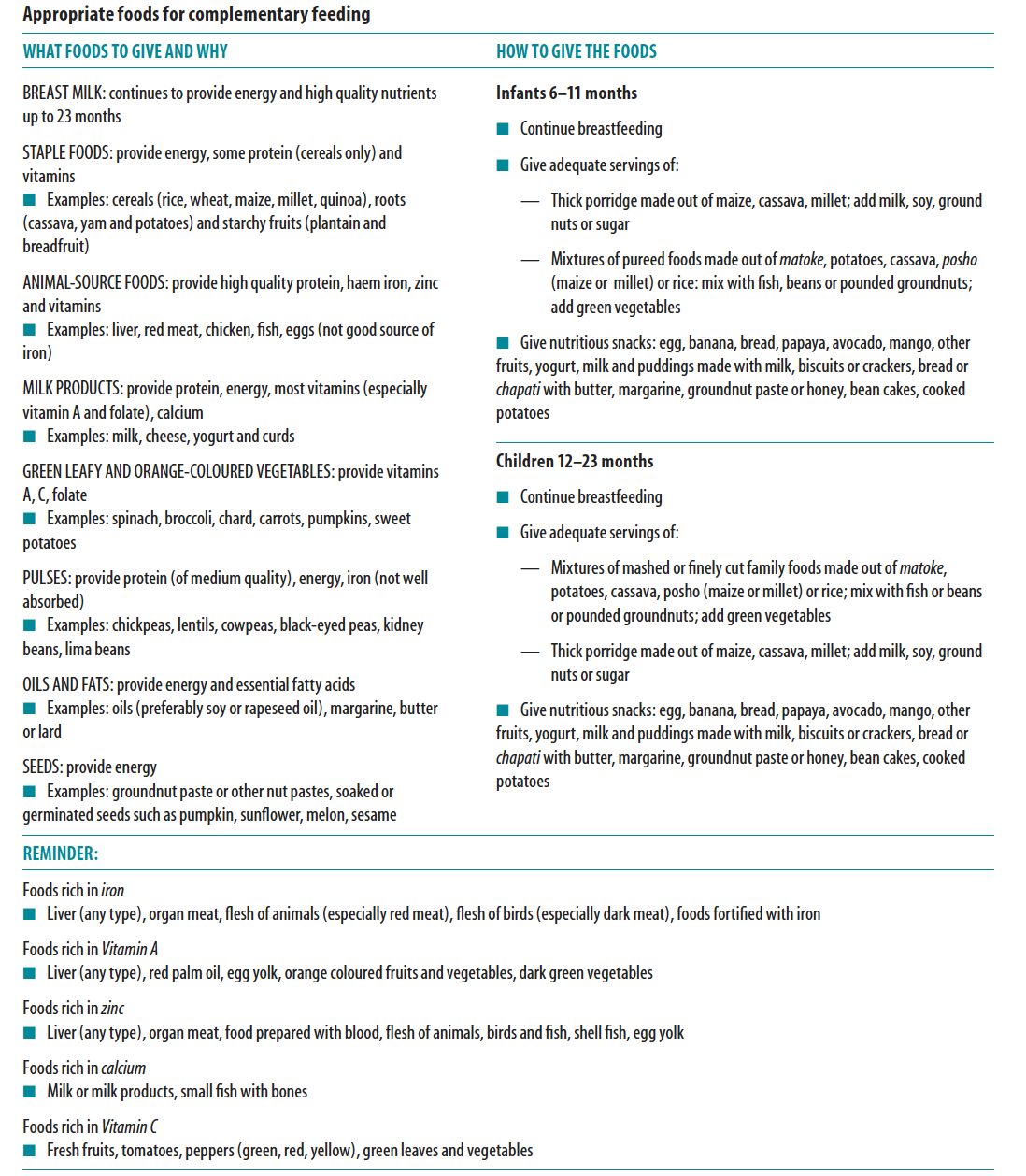


World Health Organization (WHO). Infant and young child feeding: Model chapter for textbooks for medical students and allied health professionals. Geneva: WHO Press, 2009. 15.
